# LDH-to-Albumin Ratio (LAR) in Solid Tumor Patients with Cytopenia: A Simple Biomarker to Predict Bone Marrow Metastasis

**DOI:** 10.3390/jcm14207379

**Published:** 2025-10-18

**Authors:** Tugba Cetintepe, Lutfi Cetintepe, Zeynep Guc, Ibrahim Ertekin, Onur Karaalp, Ahmet Alacacioglu

**Affiliations:** 1 Department of Hematology, İzmir Kâtip Çelebi University, Atatürk Training and Research Hospital, 35730 İzmir, Türkiye; 2 Department of Nephrology, İzmir Kâtip Çelebi University, Atatürk Training and Research Hospital, 35730 İzmir, Türkiye; lutficetintepe@gmail.com (L.C.); cabala9@gmail.com (I.E.); 3 Department of Oncology, İzmir Kâtip Çelebi University, Atatürk Training and Research Hospital, 35730 İzmir, Türkiye; zeynepgsevgen@gmail.com (Z.G.); onurkaraalp67@gmail.com (O.K.); dralacacioglu@hotmail.com (A.A.)

**Keywords:** bone marrow metastasis, solid tumor, cytopenia, inflammatory biomarkers, LAR, LDH, survival, prognostic factors

## Abstract

**Objectives**: This study evaluated the frequency of bone marrow metastasis (BMM), its associated clinical and laboratory parameters, and its effects on survival in patients with solid tumors who underwent bone marrow biopsy due to cytopenia. Furthermore, the diagnostic power of inflammatory biomarkers (LAR, NLR, PLR, HALP, and SII) in predicting BMM was systematically analyzed for the first time in a large sample. **Methods**: Data from 233 patients with solid tumors were retrospectively reviewed. Fifteen patients with therapy-related high-risk myelodysplastic syndrome and acute myeloid leukemia were excluded, leaving 218 patients included in the study. Cytopenia was categorized according to CTCAE5.0. ROC analyses were performed for the inflammatory biomarkers LAR, NLR, PLR, SII, and HALP score. **Results**: BMM was detected in 39.9% (*n* = 87) of all patients. Prostate cancer exhibited the highest incidence of BMM, while bone represented the most common site of concurrent metastasis (*p* < 0.01). Hematologic and biochemical abnormalities—including low hemoglobin, platelet, and albumin levels, along with elevated LDH—were significantly associated with BMM (*p* < 0.01 for all). Elevated inflammatory indices (LAR, PLR, and SII) also correlated with higher risk. A multivariate analysis demonstrated that LAR provided the strongest predictive value (AUC: 0.939; 95% CI: 0.901–0.955; *p* < 0.01), with an optimal cutoff of 9.21. **Conclusions**: BMM is an important condition that negatively affects survival in solid tumor patients with cytopenia. The risk of BMM especially increases in male patients and in those with high LDH and low levels of albumin, hemoglobin, and platelets. Among the evaluated inflammatory biomarkers, LAR showed the highest diagnostic performance in predicting BMM.

## 1. Introduction

Bone marrow metastasis (BMM) in solid tumors occurs when hematopoietic non-malignant cells settle in the bone marrow via the blood or lymphatic system. The presence of cancer cells in the bone marrow affects its structure, leading to hematopoietic dysfunction and cytopenia [1]. Although rare, it is highly significant in terms of disease progression [2]. Breast, lung, stomach, and prostate tumors are the primary solid tumors that tend to metastasize to the bone marrow. Bone marrow metastasis alters the stage of the disease and its prognosis, and it directly affects treatment responses, playing a decisive role in morbidity and mortality [3,4,5].

The metastasis of solid tumors to the bone marrow is not solely dependent on the characteristics of the tumor cells but is also regulated by factors related to the bone marrow microenvironment [6]. Despite numerous recent studies on molecular mechanisms, it remains unclear which tumor cells metastasize under which conditions and when these metastases will manifest clinically or remain silent as micrometastases [7,8,9,10].

In solid tumors, BMM may be asymptomatic, but unexplained prolonged cytopenias are the most common clinical findings [11]. It is quite difficult to distinguish cytopenia caused by bone marrow metastasis from primary hematological diseases and, in particular, bone marrow suppression caused by chemotherapy. Therefore, collaboration between hematologists and oncologists is essential for the early recognition and proper management of BMM.

The early diagnosis of solid tumor bone marrow metastasis is quite challenging due to the absence of specific clinical findings. BMM can be detected using sensitive imaging methods such as FDG-PET/CT and Magnetic Resonance Imaging [12,13]. However, bone marrow aspiration–biopsy remains the gold standard diagnostic method for identifying micrometastases, excluding primary hematological diseases, and determining the type of metastasis [14]. Bone marrow aspiration is a simple, rapid, and inexpensive method for detecting BMM. In clinical practice, the identification of biomarkers supporting the indication for biopsy is an important requirement. Standardizing the approach to BMM in patients with solid tumors and cytopenia may facilitate early diagnosis and spare this vulnerable patient group from unnecessary invasive procedures.

In this study, data from 233 patients with solid tumors who were referred to the hematology clinic due to the development of cytopenia and underwent bone marrow aspiration–biopsy were evaluated. The aim was to investigate the frequency of BMM in patients diagnosed with solid tumors, its associated clinical and laboratory parameters, and its effects on survival. The predictive power of inflammatory markers that could be used to predict BMM was evaluated. The aim was to better understand BMM in solid tumors and improve diagnostic, follow-up, and treatment strategies for patients who develop BMM.

## 2. Materials and Methods

Between January 2010 and January 2025, data from 233 patients with solid tumors who were followed in the oncology clinic, referred to the hematology clinic due to prolonged cytopenia, and underwent bone marrow biopsy were retrospectively reviewed. Fifteen patients were diagnosed with therapy-related high-risk myelodysplastic syndrome or acute myeloid leukemia. As these hematologic conditions could affect prognosis and blood parameters, they were excluded from the study. Ultimately, 218 patients with solid tumors who underwent bone marrow biopsy were included in the analysis.

All patient data were obtained from the hospital’s electronic medical records. Cytopenia definitions were made according to the Common Terminology Criteria for Adverse Events (CTCAE) version 5.0, and complete blood count and biochemical parameters obtained prior to bone marrow biopsy were recorded [15].

Demographic characteristics, primary tumor type, sites of metastasis, treatment protocols administered before the onset of cytopenia, and the time interval between treatment and cytopenia development were documented.

Biochemical and inflammatory markers included the lactate dehydrogenase-to-albumin ratio (LAR), neutrophil-to-lymphocyte ratio (NLR), platelet-to-lymphocyte ratio (PLR), systemic immune–inflammation index (SII), and hemoglobin/albumin/lymphocyte/platelet (HALP) score. SII was calculated as the product of the neutrophil and platelet counts divided by the lymphocyte count. The HALP score was determined using the formula hemoglobin (g/L) × albumin (g/L) × lymphocyte count (/L)/platelet count (/L).

Bone marrow biopsy fibrosis and reticulin fiber grading were performed using Giemsa, reticulin, and Masson’s trichrome histochemical stains. The degree of fibrosis was scored by a pathologist according to the modified Bauermeister Grading System guidelines [16].

## 3. Statistical Methods

Data analysis was performed using IBM SPSS Statistics, version 25.0 (IBM Corp., Armonk, NY, USA) and the Python, version 3.11 (Python Software Foundation, Wilmington, DE, USA) programming language. In Python, the statsmodels, sklearn, and matplotlib libraries were utilized for statistical analysis and data visualization (accessed on 12 September 2025). Overall survival (OS) was defined as the time from the date of solid tumor diagnosis to death or the last follow-up.

Descriptive statistics were expressed as frequency and percentage (%) for categorical variables, and as mean ± standard deviation, median (interquartile range), minimum, and maximum values for continuous variables. Differences in categorical variables between patients with and without bone BMM were evaluated using the Pearson Chi-square test.

ROC (Receiver Operating Characteristic) curve analyses were performed for the inflammatory biomarkers LAR, NLR, PLR, SII, and HALP score; the area under the curve (AUC) values were calculated, and optimal cutoff points were determined using the Youden index.

Levels of hemoglobin, neutrophils, platelets, and lactate dehydrogenase (LDH) were staged according to CTCAE version 5.0, and group differences were evaluated using the Chi-square test. The prognostic value of clinical and laboratory variables that may affect the risk of death was assessed using univariate and multivariate Cox regression models. A *p*-value < 0.05 was considered statistically significant for all tests.

## 4. Results

### 4.1. Patient Demographics and Clinical Characteristics

The study included 218 patients with a diagnosis of solid tumors who underwent bone marrow biopsy due to cytopenia. Of the patients, 63.8% were female and 36.2% were male, with an average age of 56.8 years. Fifty-eight point seven percent of the cases were under the age of 65.

Bone marrow metastasis was detected in 39.9% (*n* = 87) of all patients. The reported frequency of BMM refers to biopsy-confirmed cases; all patients in this study underwent bone marrow biopsy due to cytopenia, and the diagnosis of bone marrow metastasis was established histopathologically rather than based on pre-biopsy clinical or radiological assessment. The majority of patients included in the study had breast cancer (*n* = 80). When diseases were categorized according to primary diagnosis types, the group with the highest likelihood of bone marrow metastasis was prostate cancer (*p* < 0.01). Bone marrow metastasis was detected in 82.4% of prostate cancer patients who underwent bone marrow biopsy due to cytopenia. Prostate cancer was followed by breast cancer (53.8%) and lung cancer (50%). BMM was detected in very few cases of colon cancer, gynecological cancers, and glioblastoma. Among the 218 patients, 101 (46.3%) had radiologically confirmed bone metastases, and 117 (53.7%) did not. Bone marrow metastasis was detected in 73 of 101 patients (72.3%) with bone metastases and in 14 of 117 patients (12.0%) without bone metastases. This association was statistically significant (Table 1).

### 4.2. Laboratory Parameters, Imaging Findings, and Treatment-Related Factors Associated with Bone Marrow Metastasis

Among the various metastatic sites in solid tumors, bone was found to be the most common site of concurrent metastasis (46.3%). Notably, 73% of patients with bone metastasis also had bone marrow metastasis, a statistically significant association (*p* < 0.01). Bone metastases were most commonly accompanied by liver and lymph node metastases, respectively.

Pre-biopsy hemogram and biochemical parameters were classified according to CTCAE v5.0 and their relationship with BMM was examined (Table 2). Low hemoglobin and platelet levels and high LDH levels were significantly associated with BMM (each *p* < 0.01). Furthermore, patients with low albumin levels had a significantly higher rate of BMM (*p* < 0.01).

Patients with increased bone marrow fibrosis (myelofibrosis grades 1, 2, or 3) showed a higher rate of BMM (*p* = 0.0009).

There were 77 patients who underwent 18F-FDG PET/CT imaging concurrently with bone marrow biopsy. Bone marrow metastasis was detected in the biopsies of 35 of these patients and was also identified on PET/CT imaging. However, PET/CT imaging was normal in two patients, with bone marrow metastasis detected by biopsy.

In terms of treatment modalities, the risk of bone marrow metastasis was significantly reduced in patients receiving chemotherapy (HR = 0.327, *p* < 0.01), and longer duration of chemotherapy emerged as a risk-reducing factor (HR = 0.812, *p* = 0.008). No significant change in risk was observed in patients receiving hormonal therapy and immunotherapy. A higher proportion of patients with bone marrow metastasis had also received radiotherapy, which may be related to palliative treatment for bone metastases. Although the hazard ratio for radiotherapy was 1.535, this association was not statistically significant (*p* = 0.171).

### 4.3. Diagnostic Power of Inflammatory Biomarkers in Predicting BMM

Table 3 summarizes the distributions of inflammatory and nutritional indices (median, interquartile range) in patients with and without biopsy-proven bone marrow metastases, along with *p*-values calculated using the Mann–Whitney U test. These indices were evaluated as potential pre-biopsy markers to predict the likelihood of bone marrow metastases before histological confirmation. As shown, SII, PLR, HALP, and LAR differed significantly between groups, whereas NLR showed no statistically significant difference.

**Figure 1:**
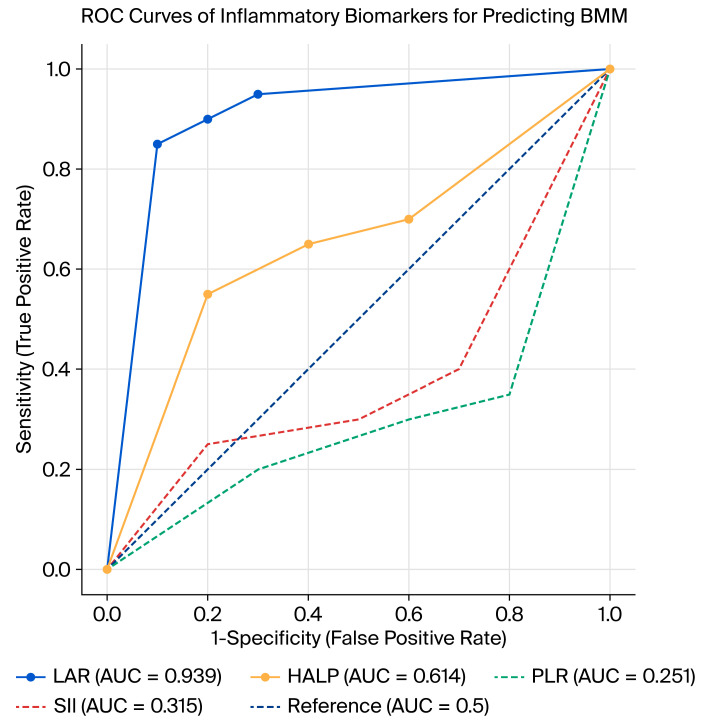
ROC curves of inflammatory biomarkers for predicting BMM.

ROC analysis was performed to evaluate the diagnostic performance of LAR, HALP, NLR, PLR, and SII in predicting BMM (Figure 1). The results demonstrated that only LAR and HALP showed statistically significant diagnostic value, whereas NLR, PLR, and SII did not demonstrate significant predictive power (Table 4).

LAR had the highest diagnostic accuracy with an AUC of 0.939 (95% CI: 0.908–0.971; *p* < 0.01). The optimal cutoff point was calculated as 9.21, yielding a sensitivity of 85%, specificity of 86%, positive predictive value (PPV) of 79%, and negative predictive value (NPV) of 90%.

HALP showed moderate but significant predictive ability with an AUC of 0.614 (95% CI: 0.536–0.693; *p* = 0.004). The best cutoff value was 1.35, providing a sensitivity of 74%, specificity of 62%, PPV of 56%, and NPV of 78%.

NLR (AUC: 0.501; 95% CI: 0.424–0.579; *p* = 0.973), PLR (AUC: 0.251; 95% CI: 0.184–0.319; *p* < 0.01), and SII (AUC: 0.315; 95% CI: 0.243–0.386; *p* < 0.01) did not show clinically useful diagnostic performance. Although PLR and SII reached statistical significance, their AUC values were <0.5, indicating inverse or non-meaningful discriminative performance rather than true diagnostic utility. Among the evaluated indices, only LAR and HALP showed meaningful diagnostic accuracy (AUC > 0.5, *p* < 0.05), whereas NLR, PLR, and SII did not demonstrate positive discriminative ability despite statistical significance (AUC < 0.5).

In summary, LAR emerged as the most reliable biomarker for predicting BMM, while HALP had limited but significant diagnostic utility. Other inflammatory indices (NLR, PLR, and SII) did not demonstrate sufficient discriminative ability and therefore may not be clinically useful in this context.

To evaluate whether the diagnostic accuracy of LAR was driven by prostate cancer patients, a sensitivity analysis was conducted. The ROC analysis performed in the subgroup excluding prostate cancer patients demonstrated that LAR retained excellent diagnostic performance, with an AUC of 0.944 (95% CI: 0.915–0.973; *p* < 0.01) (Table 5). The optimal cutoff was 9.0, yielding a sensitivity value of 84%, specificity of 86%, PPV of 77%, and NPV of 90%. These findings were highly consistent with the results for the entire cohort (AUC: 0.939, 95% CI: 0.901–0.955; cutoff: 9.21; sensitivity: 85%, specificity: 86%). Thus, the predictive power of LAR was not confined to prostate cancer cases but was robust across different tumor types.

### 4.4. Bone Marrow Metastasis and Overall Survival

**Figure 2:**
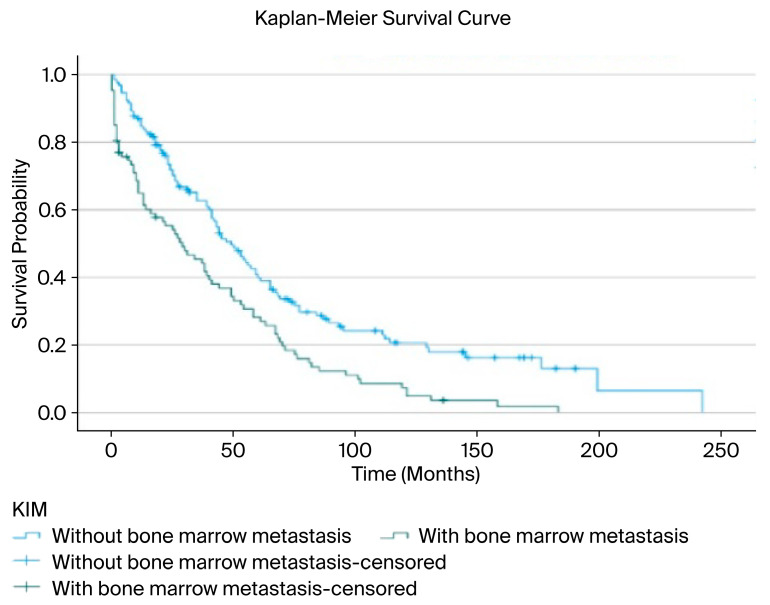
Overall survival associated with bone marrow metastasis.

In our study, the relationship between the presence of BMM and overall survival (OS) was analyzed using the Kaplan–Meier method. The median overall survival for the entire patient cohort was determined to be 41 months (95% CI: 33.9–48.0). The median survival was 49 months (95% CI: 39.5–58.5) in the group without BMM, compared to 29 months (95% CI: 14.5–43.5) in the group with BMM. These findings clearly indicate a significantly negative impact of BMM on patient survival (Figure 2).

A survival analysis demonstrated a significant difference between patients with and without bone marrow metastasis. Since the survival curves crossed at approximately 36 months, a piecewise analysis was performed. During the first 36 months, patients without bone marrow metastasis had significantly longer survival compared with those with metastasis (mean 18.7 vs. 11.1 months; median 20 vs. 9 months; log-rank χ^2^ = 11.134, *p* < 0.01). After 36 months, the difference remained statistically significant, though less pronounced (mean 107.5 vs. 78.0 months; median 77 vs. 68 months; log-rank χ^2^ = 5.402, *p* = 0.020) (Table 6).

### 4.5. Risk Factors Associated with Bone Marrow Metastasis: Cox Regression Analysis Results

In our study, factors associated with the development of BMM were evaluated using Cox regression analysis through both univariate and multivariate models. In the univariate analyses, male gender, low hemoglobin, low albumin, and high LDH, LAR, PLR, and SII were found to have significant effects, whereas NLR was not associated with BMM. HALP showed only borderline significance.

According to the multivariate Cox regression model, male gender (HR: 2.024; 95% CI: 1.215–3.373; *p* = 0.007), elevated LAR (HR: 5.271; 95% CI: 2.611–10.641; *p* < 0.01), and the presence of concomitant bone metastasis (HR: 2.355; 95% CI: 1.262–4.393; *p* = 0.007) were identified as independent predictors for bone marrow metastasis (Table 7).

Since LAR is mathematically derived from LDH and albumin, potential collinearity was assessed by constructing alternative Cox models (see Supplementary Tables S1 and S2). In these analyses, albumin consistently remained an independent prognostic factor, and LAR also retained its predictive value when analyzed separately.

These findings are consistent with the ROC analysis results, which demonstrated that LAR was the only biomarker with strong diagnostic performance, whereas HALP had limited value and NLR, PLR, and SII did not show meaningful predictive ability.

## 5. Discussion

The aim of this study was to evaluate the frequency of BMM and its associated clinical parameters in patients with solid tumors who developed cytopenia. In our study, only patients who underwent bone marrow biopsy due to unexplained cytopenia were included. This selection was intentional, as cytopenia is one of the most common clinical manifestations of bone marrow infiltration. In clinical practice, bone marrow biopsy is not routinely performed in all cancer patients but is primarily indicated when cytopenia raises suspicion of bone marrow involvement. Therefore, this subgroup represents the population at highest risk of bone marrow metastasis. By focusing on cytopenic patients, our study provides clinically meaningful insights into the diagnostic and prognostic relevance of inflammatory and nutritional biomarkers in a group where the decision to perform a biopsy is both most frequent and most critical. This approach may help clinicians better stratify risk, reduce unnecessary procedures, and optimize early management strategies for patients with advanced solid tumors.

A key strength of our study is that all 218 patients who underwent bone marrow biopsy had a confirmed diagnosis of a primary solid tumor. Only patients with solid tumors who developed cytopenia and subsequently underwent bone marrow biopsy were included in the study. BMM was identified in 39.9% of the 218 cytopenic solid tumor patients. The majority of the included patients had breast cancer (*n* = 80). However, when categorized according to the type of primary tumor, prostate cancer emerged as the malignancy with the highest likelihood of BMM. Among prostate cancer patients who underwent bone marrow biopsy due to cytopenia, 82.4% were found to have BMM. This was followed by breast, lung, and gastric cancers, each with BMM rates of around 50%, consistent with previous literature [11,17,18,19].

In our cohort, prostate cancer patients had the highest incidence of BMM. This rate was considerably higher than reported in previous studies, likely because many of our patients were in the post-treatment metastatic stage [20]. These findings suggest that in prostate cancer patients with prolonged and severe cytopenia, BMM should be strongly suspected.

Gender-based differences also revealed noteworthy findings. The significantly increased risk of BMM in male patients may reflect sex-specific differences in hormonal, immunological, and tumor biology factors. This observation aligns with reports in the literature suggesting that male gender is associated with more aggressive disease courses in certain solid tumors [21]. Although no statistically significant difference was observed in baseline gender distribution between patients with and without bone marrow metastases (Table 1, *p* = 0.680), gender became a significant predictor in the multivariate Cox regression model (Table 7). This indicates that, while gender alone did not differ significantly at baseline, male sex was independently associated with the development of bone marrow metastases after adjustment for potential confounding variables.

When our patients with BMM were evaluated for concurrent metastasis to other organs, the presence of bone metastasis emerged as an important finding. BMM was detected in 73 of 101 patients with solid tumors and bone metastasis. Considering the interaction between tumor cells and the bone microenvironment, this result is not surprising from a pathophysiological perspective [22]. Although some studies have mentioned this association in the literature, the number of reported cases is limited [19,23]. Our study has shown that in patients with solid tumors and bone metastases, the presence of concomitant cytopenia increases the likelihood of bone marrow metastasis.

Myelofibrosis may develop as a stromal reaction to metastatic cells in the bone marrow, disrupting the hematopoietic microenvironment and worsening cytopenia, survival, and treatment tolerance [24,25,26]. In our cohort, BMM was more common in patients with higher grades of bone marrow fibrosis, suggesting that fibrosis evaluation may provide prognostic and therapeutic insights.

Cytopenia may occur for various reasons during the follow-up of solid tumors. The most common cause is chemotherapy-related myelosuppression, while concomitant primary hematological diseases, malnutrition, infections, and autoimmune mechanisms may also be causes of cytopenia. In patients referred to hematology, all of these conditions were reviewed at the initial presentation; however, when no identifiable cause was found, a bone marrow biopsy was performed. No significant relationship was found between neutrophil levels and BMM. Decreases in hemoglobin and platelet levels were found to be associated with bone marrow metastasis, similar to the literature [11,27,28,29].

Elevated LDH levels, reflecting tumor metabolism and invasiveness, have frequently been reported in patients with BMM and were among the strongest markers in our study (*p* < 0.01) [26,30,31,32]. Low albumin levels have previously been linked to increased tumor burden and distant metastasis [33,34,35,36]. However, to our knowledge, no prior studies have directly examined the association between albumin and BMM. Our study is the first to demonstrate a strong relationship between hypoalbuminemia and the development of BMM (*p* < 0.01).

The strong association between LAR and bone marrow metastasis observed in our study can be explained by the dual biological role of its components. Elevated LDH levels are indicative of tumor burden, glycolytic reprogramming, and hypoxia-driven aggressiveness, which are hallmarks of advanced malignancy and metastatic spread. In contrast, albumin levels reflect both nutritional status and systemic inflammation. Hypoalbuminemia, commonly seen in cancer patients, results from cytokine-mediated catabolism (e.g., IL-6, TNF-α), reduced protein synthesis, and increased vascular permeability. Low albumin thus signals malnutrition and an impaired host response, conditions that promote tumor progression and bone marrow infiltration. By integrating high LDH and low albumin, LAR captures both tumor aggressiveness and host vulnerability, making it a comprehensive predictor of bone marrow metastasis. This mechanistic rationale supports our finding that LAR outperformed other inflammatory indices in diagnostic accuracy [30,37].

One of the original contributions of this study is the evaluation of the diagnostic performance of various inflammatory biomarkers in predicting BMM through ROC curve analyses. Among these, LAR emerged as the biomarker with the highest diagnostic accuracy, with an AUC value of 0.939, indicating that it increases the risk of BMM approximately sixfold. Similarly, markers reflecting systemic inflammation such as the platelet-to-lymphocyte ratio (PLR) and systemic immune–inflammation index (SII) were also found to be significantly associated with BMM. These findings support the hypothesis that inflammation may act as a trigger and sustainer of the metastatic process. Although prior studies have examined the prognostic value of LAR in various solid tumors, none have specifically evaluated its utility in predicting BMM [37]. The HALP score has previously been used to predict survival, and its association with BMM in this study suggests that the combined assessment of hematologic and nutritional status may provide meaningful insights regarding BMM.

Currently, 18F-FDG PET/CT is one of the commonly used imaging modalities for assessing systemic metastases. In our study, 77 patients underwent FDG-PET/CT imaging concurrently with bone marrow biopsy. Among these, BMM was detected both by biopsy and PET/CT in 35 patients. In two cases, metastasis was found on biopsy but not detected on PET/CT imaging. Our results align with the literature, demonstrating that FDG-PET/CT has high sensitivity for detecting BMM [12,38]. However, rare cases of microscopic or metabolically inactive metastases may be missed by imaging. These data indicate that in solid tumor patients with cytopenia, even if PET/CT results are normal, BMM cannot be ruled out, and bone marrow biopsy should be considered when clinical suspicion exists.

When evaluating treatment-related findings, it is noteworthy that patients receiving chemotherapy had a lower risk of developing BMM. This may reflect the suppressive effect of systemic therapies on micrometastatic disease. Furthermore, longer treatment duration emerged as a protective factor, suggesting that sustained systemic suppression may limit disease progression. Other treatment modalities did not significantly impact BMM development.

Kaplan–Meier survival analyses revealed a significantly negative effect of BMM presence on overall survival. While the median survival was approximately 49 months in patients without BMM, it decreased to 29 months in those with BMM. This finding supports the notion that BMM is not only a marker of advanced disease stage, but also an independent prognostic factor. Our results are consistent with previously reported survival data in the literature [27].

One of the strengths of this study is the analysis of a large BMM cohort confirmed by biopsy and the multidimensional analysis of clinical, laboratory, and imaging data. Additionally, the first-time combined evaluation of biomarkers such as HALP, LAR, and SII contributes uniquely to this study. The study had some limitations. It was a retrospective study conducted at a single center. Patients who were unable to undergo invasive procedures due to poor general condition but were highly suspected of having bone marrow metastasis were excluded from the study. Although all our patients had a diagnosis of solid tumors, the patient group was heterogeneous when considering the primary disease groups. Well-designed, multicenter, prospective studies are needed in this regard.

## 6. Conclusions

In this study, the frequency of BMM in patients with solid tumors who underwent bone marrow biopsy due to cytopenia, as well as its associated clinical parameters and effects on survival, were evaluated. Additionally, the diagnostic utility of inflammatory biomarkers (LAR, NLR, PLR, HALP, and SII) in predicting BMM was systematically analyzed for the first time in this large cohort. The findings demonstrated that the presence of BMM significantly shortened median survival and that certain variables were significant risk factors for BMM development, including male gender, elevated LDH levels, low hemoglobin and albumin levels, increased inflammatory indices (LAR, PLR, and SII), and concomitant bone metastasis. A multivariate analysis determined the prognostic power of LAR in predicting BMM (AUC: 0.939; 95% CI: 0.901–0.955; *p* < 0.01). The optimal cutoff point for LAR was calculated as 9.21. The high diagnostic accuracy of the LAR biomarker in predicting BMM may be beneficial in reducing unnecessary biopsies or planning early intervention in these patients. The routine use of these biomarkers in clinical decision-making processes may facilitate the management of patients with advanced-stage cancer.

## Figures and Tables

**Table 1:** Comparison of patient characteristics according to bone marrow metastasis.

Variable	Overall (*n* = 218)	Bone Marrow Metastasis		*p*
		No 131 (60.1%)	Present 87 (39.9%)	
Age (years), mean ± SD	56.8	56.6	56.8	0.067
Gender, *n* (%)				
Male	79 (36.2)	48 (60.7)	31 (39.2)	0.680
Female	139 (63.8)	83 (59.7)	56 (40.2)	
Solid Tumor *n* (%)				
Breast	80 (36.6)	37 (46.2)	43 (53.8)	<0.01
Gastric	28 (12.8)	16 (57.1)	12 (42.9)	
Other	28 (12.8)	22 (78.6)	6 (21.4)	
Colon	24 (11.0)	23 (95.8)	1 (4.2)	
Prostate	17(7.7)	3 (17.6)	14 (82.4)	
Gynecological	15 (6.8)	15 (100)	0 (0.0)	
Lung	14 (6.4)	7 (50.0)	7 (50.0)	
Head and Neck	7 (3.2)	4 (57.1)	3 (42.9)	
Glioblastoma	3 (1.3)	3 (100)	0 (0.0)	
Malign Melanoma	2 (0.9)	1 (50.0)	1 (50.0)	
Solid Tumor Metastasis *n*				
Bone	101	28	73	0.01
Lymph Node	66	38	28	
Liver	63	29	34	
Lung	40	23	12	
Brain	15	6	9	
Adrenal	5	2	3	
Albumin, *n* (%)				
Low	93(42.6)	16 (17.2)	77 (82.7)	<0.01
Normal	125 (57.4)	115 (92)	10 (8)	
Lymphocyte *n* (%)				
Low	136 (62.3)	92 (67.6)	44 (32.3)	0.0053
Normal	82 (37.6)	39 (47.5)	43 (52.4)	
Overall survival (median months)	41	49	29	0.0011
Follow-up time (mean ± SD) months		61.99 ± 55.71 (1–242)	39.01 ± 41.30 (1–183)	<0.05

**Table 2:** Association of laboratory findings classified according to CTCAE with bone marrow metastasis.

Variable	Bone Marrow Metastasis		*p*
	No 131 (60.1%)	Present 87 (39.9%)	
Neutrophils,			
Grade 0	77 (58.8)	59 (67.8)	
Grade 1	16 (12.2)	14 (16.1)	
Grade 2	29 (22.1)	11 (12.6)	0.219
Grade 3	6 (4.6)	1 (1.1)	
Grade 4	3 (2.3)	2 (2.3)	
Hemoglobin,			
Grade 0	53 (40.5%)	3 (3.4%)	<0.01
Grade 1	23 (17.6)	14 (16.1)	
Grade 2	49 (37.4)	47 (54)	
Grade 3	5 (3.8)	18 (20.7)	
Grade 4	1 (0.8)	5 (5.7)	
Platelets,			
Grade 0	38 (29.0)	10 (11.5)	
Grade 1	45 (34.4)	16 (18.4)	
Grade 2	31 (23.7)	16 (18.4)	<0.01
Grade 3	14 (10.7)	22 (25.3)	
Grade 4	3 (2.3)	23 (26.4)	
Lactate Dehydrogenase (LDH),			
Grade 0	85 (64.9)	9 (10.3)	<0.01
Grade 1	43 (32.8)	36 (41.4)	
Grade 2	3 (2.3)	26 (29.9)	
Grade 3	0 (0.0)	14 (16.1)	
Grade 4	0 (0.0)	2 (2.3)	

**Table 3:** Comparison of inflammatory/nutritional indices according to bone marrow metastases status.

Parameter	No BMM (*n* = 131) Median (IQR)	BMM (*n* = 87) Median (IQR)	Mann–Whitney U	*p*-Value
SII	249.0 (334.2)	139.3 (178.2)	3585.5	<0.01
PLR	113.8 (116.7)	44.0 (75.0)	2864.5	<0.01
NLR	2.70 (4.33)	2.67 (3.38)	5682.0	0.971
HALP	3.52 (3.83)	5.26 (7.16)	4394.0	0.004
LAR	5.80 (3.15)	19.2 (22.4)	691.0	<0.01

**Table 4:** Diagnostic performance of inflammatory/nutritional biomarkers for predicting bone marrow metastases (ROC analysis).

Biomarker	AUC (95% CI)	*p*-Value	Cutoff	Sensitivity (%)	Specificity (%)	PPV (%)	NPV (%)
LAR	0.939 (0.908–0.971)	<0.01	9.21	85	86	79	90
HALP	0.614 (0.536–0.693)	0.004	1.35	74	62	56	78
NLR	0.501 (0.424–0.579)	0.973	–	–	–	–	–
PLR	0.251 (0.184–0.319)	<0.01	–	–	–	–	–
SII	0.315 (0.243–0.386)	<0.01	–	–	–	–	–

**Table 5:** Diagnostic performance of LAR in predicting BMM before and after excluding prostate cancer patients.

Group	Cutoff	AUC (95% CI)	*p*-Value	Sensitivity (%)	Specificity (%)	PPV (%)	NPV (%)
Entire cohort (*n* = 218)	9.21	0.939 (0.901–0.955)	<0.01	85.0	86.0	79.0	90.0
Excluding prostate cancer (*n* = 201)	9.00	0.944 (0.915–0.973)	<0.01	83.6	85.9	77.2	90.2

**Table 6:** Time-dependent survival analysis according to bone marrow metastasis status.

Period	Bone Marrow Metastasis	Mean Survival (Months) [95% CI]	Median Survival (Months) [95% CI]	Log-Rank χ^2^	*p*-Value
0–36 months	Absent	18.7 (15.9–21.5)	20 (15.3–24.7)	11.134	<0.01
	Present	11.1 (8.1–14.1)	9 (5.2–12.8)		
>36 months	Absent	107.5 (89.4–125.7)	77 (56.8–97.2)	5.402	0.020
	Present	78.0 (65.7–90.2)	68 (59.7–76.3)		

**Table 7:** Evaluation of factors associated with bone marrow metastasis using the Cox regression model.

	Univariate			Multivariate		
Variable	HR	95% CI	*p*	HR	95% CI	*p*
Age	1.012	0.996–1.029	0.147	1.018	0.997–1.039	0.086
Age (<60 versus ≥60 years)	1.131	0.730–1.751	0.582			
Male sex	1.729	1.099–2.720	0.018	2.024	1.215–3.373	0.007
Laboratory category						
Neutrophils	1.170	0.757–1.806	0.480			
Lymphocytes	1.674	1.096–2.558	0.017			
Hemoglobin	0.296	0.177–0.496	<0.01			
Platelets	0.462	0.243–0.876	0.018			
LDH	3.797	2.466–5.846	<0.01			
Albumin	0.113	0.058–0.219	<0.01			
Index category						
LAR	6.078	3.347–11.039	<0.01	5.271	2.611–10.641	<0.01
NLR	0.960	0.629–1.464	0.849			
PLR	0.531	0.338–0.832	0.006	1.190	0.638–2.221	0.585
SII	0.504	0.324–0.782	0.002	0.628	0.361–1.093	0.100
HALP	1.462	0.950–2.250	0.084			
Site of metastasis before bone marrow involvement						
Bone	4.477	2.512–7.979	<0.01	2.355	1.262–4.393	0.007
CNS	1.062	0.529–2.133	0.866			
Lymph node	1.456	0.917–2.301	0.111			
Liver	1.378	0.893–2.128	0.148			
Lung	0.791	0.429–1.457	0.452			
Adrenal	4.339	1.328–14.177	0.015	1.232	0.349–4.347	0.746
Treatment modality						
None		Reference				
Chemotherapy	0.327	0.197–0.542	<0.01			
Hormonotherapy	1.180	0.604–2.305	0.628			
Immunotherapy	0.859	0.199–3.698	0.838			
Chemotherapy duration (months)	0.812	0.696–0.947	0.008			

Abbreviations: CNS, central nervous system; CI, confidence interval; HALP, hemoglobin/albumin/lymphocyte/platelet score; HR, hazard ratio; LAR, lactate dehydrogenase-to-albumin ratio; LDH, lactate dehydrogenase; NLR, neutrophil-to-lymphocyte ratio; PLR, platelet-to-lymphocyte ratio; SII, systemic inflammation index; WBC, white blood cell.

## Data Availability

Data and SPSS metadata are available from the corresponding author upon request. Analyses were performed using IBM SPSS Statistics v25.0 and Python v3.11.

## Supplementary Materials

The following supporting information can be downloaded at https://www.mdpi.com/article/10.3390/jcm14207379/s1, Table S1: Multivariable Cox regression analysis for bone marrow metastasis (Model 2,3). Table S2: Multivariable Cox regression analysis for bone marrow metastasis (Model 4,5).

## Abbreviations

The following abbreviations are used in this manuscript:

BMM	Bone marrow metastasis
CTCAE	Common Terminology Criteria for Adverse Events
LAR	Lactate dehydrogenase-to-albumin ratio
NLR	Neutrophil-to-lymphocyte ratio
PLR	Platelet-to-lymphocyte ratio
SII	Systemic immune–inflammation index
OS	Overall survival
ROC	Receiver Operating Characteristic
AUC	Area under the curve
PPV	Positive predictive value
NPV	Negative predictive value
LDH	Lactate dehydrogenase
HR	Hazard ratio
HALP	Hemoglobin/albumin/lymphocyte/platelet score
