# Supplementary material for: LDH-to-Albumin Ratio (LAR) in Solid Tumor Patients with Cytopenia: A Simple Biomarker to Predict Bone Marrow Metastasis
Source: J Clin Med. 2025 Oct 18;14(20):7379. doi: 10.3390/jcm14207379 (PMC12564924; doi:10.3390/jcm14207379)
Supplement: Supplementary file 1 [file jcm-14-07379-s001.zip › jcm-3900717-supplementary.pdf]

Table S1: Multivariable Cox regression analysis for bone marrow metastasis model 2-3

	Model 2			Model 3		
**Variables**	**HR**	**95% CI**	***p***	**HR**	**95% CI**	***p***
Age	1.026	1.006-1.047	**0.012**	1.011	0.993-1.029	0.218
Male sex	1.628	0.973-2.725	0.063	2.011	1.215-3.329	**0.007**
BM RLD Group				0.648	0.378-1.111	0.115
Laboratory						
Hemoglobin				0.843	0.715-0.995	**0.043**
Platelets				1.000	0.997-1.003	0.970
LDH				1.001	1.001-1.002	**0.001**
Albumin						
Indices						
LAR						
NLR						
PLR						
SII						
HALP	1.013	1.001-1.026	**0.049**			
Indices category						
LAR	4.305	2.232-8.302	**<0.001**			
NLR						
PLR						
SII						
HALP						
Metastasis before bone marrow involvement				2.211	1.061-4.604	0.034
Sites of metastasis before bone marrow involvement						
Bone	3.557	1.889-6.700	**<0.001**			
Adrenal						
Treatment modality						
None		Reference				
ChemoT	0.274	0.155-0.483	**<0.001**			

HormonoT	0.453	0.215-0.956	**0.038**			
ImmunoT	1.082	0.233-5.017	0.920			
ChemoT duration (months)				0.834	0.712-0.978	**0.025**

Abbreviations: BM, bone marrow; ChemoT, chemotherapy; CNS, central nervous system; CI, coefficient; HormonoT, hormonal therapy; HALP, hemoglobin/albumin/lymphocyte/platelet score; HR, hazard ratio; LAR, LDH-to-albumine ratio; LDH, lactate dehydrogenase; NLR, neutrophile-to-lymphocyte ratio; ImmunoT, immunotherapy; PLR, platelet-to-lymphocyte ratio; RLD, reticular fiber density; SII, systemic inflammation index; WBC, white blood cell.

Table S2: Multivariable Cox regression analysis for bone marrow metastasis model 4-5

	Model 4			Model 5		
**Variables**	**HR**	**95% CI**	***p***	**HR**	**95% CI**	***p***
Age	1.024	1.005-1.043	**0.015**	1.023	1.001-1.045	**0.037**
Male sex	1.795	1.057-3.046	**0.030**	1.832	1.060-3.165	**0.030**
BM RLD Group				0.630	0.352-1.128	0.120
Hemoglobin (per 1 g/dL decrease)	1.144	0.963-1.359	0.125	1.121	0.939-1.337	0.205
Platelets				1.002	0.999-1.005	0.271
LDH (per 100 U/L increase)	1.038	1.011-1.065	**0.005**	1.027	0.998-1.057	0.070
Albumin (per 1 g/dL decrease)	1.096	1.054-1.139	**<0.001**	1.095	1.050-1.143	**<0.001**
Indices						
LAR						
NLR						
PLR						
SII	0.999	0.999-1.0	0.213			
HALP						
Indices category						
LAR				2.596	1.208-5.577	**0.014**
NLR						
PLR						
SII				0.616	0.365-1.039	0.069
HALP						
Sites of metastasis before bone marrow involvement						
Bone	2.598	1.400-4.820	**0.002**	2.682	1.391-5.170	**0.003**
Adrenal	1.126	0.321-3.953	0.853	1.175	0.330-4.191	0.803
Treatment modality						
None		Reference			Reference	
ChemoT	0.283	0.156-0.515	**<0.001**	0.279	0.141-0.553	**<0.001**

HormonoT	0.476	0.210-1.078	0.075	0.380	0.161-0.897	**0.027**
ImmunoT	0.547	0.116-2.590	0.447	0.671	0.124-3.636	0.644
ChemoT duration (months)				0.992	0.894-1.102	0.887

Abbreviations: BM, bone marrow; ChemoT, chemotherapy; CNS, central nervous system; CI, coefficient interval; HormonoT, hormonal therapy; HALP, hemoglobin/albumin/lymphocyte/platelet score; HR, hazard ratio; LAR, LDH-to-albumine ratio; LDH, lactate dehydrogenase; NLR, neutrophile-to-lymphocyte ratio; ImmunoT, immunotherapy; PLR, platelet-to-lymphocyte ratio; RLD, reticular fiber density; SII, systemic inflammation index; WBC, white blood cell.
